# Boric Acid Prevents MMC- and H₂O₂-Related DNA Damage: Evidence from Cytogenetic and Comet Assays

**DOI:** 10.1007/s12011-025-04704-z

**Published:** 2025-06-21

**Authors:** Fahima Hamoud Moussa, Ece Akbas, Deniz Yuzbasioglu, Fatma Unal

**Affiliations:** 1 Department of Biology, Graduate School of Natural and Applied Science, Gazi University, Ankara, Türkiye; 2 Department of Biology, Faculty of Science, Gazi University, Ankara, Türkiye

**Keywords:** Boric acid, Antigenotoxic effect, Chromosomal aberration test, Sister chromatid exchange test, Cytokinesis block micronucleus cytome test, Comet test, Human lymphocytes

## Abstract

Boron compounds, such as boric acid(BA-H_3_BO_3_), have been utilized as potential candidates for modulating various biological functions owing to their specific characteristics, such as low toxicity, interaction with biomolecules, and possible roles as antigenotoxic and anticancer agents. On the other hand, mitomycin-C(MMC), a chemotherapeutic drug used for several cancers, may induce genetic damage in the healthy cells of cancer patients. Therefore, this study evaluated whether BA (0.25–2.5 µg/mL) generates protective potential against MMC-induced DNA and chromosome damage. After human lymphocytes were exposed to MMC and BA alone and in combination (BA + MMC), genotoxic and/or mitigating effects were evaluated using chromosomal aberration (CAs), sister chromatid exchange (SCE) (24 and 48 h), and cytokinesis-block micronucleus cytome (48 h) tests. The ameliorative potential of BA against hydrogen peroxide(H_2_O_2_)-induced DNA damage was also assessed using a comet assay (1 h). MMC significantly increased (*p* < 0.05) the frequency of abnormal cells, CA/cell, SCE/cell, micronucleus, and nuclear buds and decreased (*p* < 0.05) the mitotic index compared to the control. However, BA alone did not induce any significant alterations in the incidence of these aberrations. In addition, all the combined treatments of BA + MMC significantly ameliorated (*p* < 0.05) all of these indices against MMC. In the comet assay, BA significantly diminished (*p* < 0.05) the tail intensity (%DNA) against H_2_O_2_. These results revealed that BA does not induce significant genotoxic effects. Moreover, it may exert chemopreventive potential against MMC- and H₂O₂-induced genetic damage. These findings suggest that boric acid is safe and effective at low concentrations in food, medicine, and healthcare applications.

## Introduction

Recent investigations into the biological effects of boron (B), a metalloid element, have demonstrated its significance as a critical trace element across a broad range of organisms, including bacteria, fungi, plants, algae, animals, and humans, all of which require only minimal quantities of this compound [1]. Owing to its unique physical and chemical properties, boron and boron-containing compounds (BCCs) have gathered considerable attention, particularly in the pharmaceutical industry [2, 3]. The beneficial health effects of BCCs are linked to various metabolic processes, as evidenced in studies involving animals, plants, and epidemiological cohorts. Naturally, boron exists in oxygen-bound forms such as borates. Examples include borax, boric acid, colemanite, and ulexite [4, 5].

BCCs have become especially prominent in targeted cancer therapies following the development of boron neutron capture therapy (BNCT) [6, 7]. This technique, commonly used to treat glioblastoma- an aggressive brain tumor- selects malignant cells while minimizing damage to adjacent healthy tissues [8, 9]. Over the past two decades, advances in organoboron chemistry have facilitated the integration of boron functional groups into pharmaceutical compounds [10]. Several BCCs have been approved by the FDA, including bortezomib (Velcade), crisaborole (Eucrisa), ixazomib (Ninlaro), tavaborole (Kerydin), and vaborbactam (in combination with meropenem in Vabomere), for the treatment of specific cancers and inflammatory diseases, demonstrating advantages over analogous boron-free agents [11]. Approximately ten additional compounds are currently undergoing clinical trials [12]. A recent study demonstrated the efficacy of BNCT as a viable therapeutic option for patients with primary or recurrent angiosarcoma or malignant melanoma, showing a favorable safety profile and high response rates [7].

Boric acid (H₃BO₃), a weak Lewis acid and the most common form of borates [13] is the predominant boron species identified in biological systems due to its water solubility [14]. At physiological pH, boric acid readily forms tetracoordinate complexes with organic molecules such as amino acids, hydroxy acids, carbohydrates, nucleotides, and vitamins, enabling its involvement in diverse biological processes. It influences the activity of several enzymes, acting either as an inhibitor or stimulator and is involved in numerous metabolic pathways [5, 15, 16]. Notably, boric acid modulates enzymes such as proteases, peptidases, and nitric oxide synthase, which play critical roles in inflammation, immune regulation, metabolism, and oncogenic signaling [17, 18]. It also contributes to cell structure, membrane function, development, and replication [19, 20]. In humans and higher animals, boric acid significantly impacts bone metabolism by modulating calcium, magnesium, and vitamin D levels and influencing brain function and lipid metabolism [3, 9]. Among postmenopausal women, boric acid has been found to regulate steroid hormones and exhibit antioxidant properties [13, 21]. Furthermore, it may serve a protective role during apoptosis by stabilizing mitochondrial membrane potential and modulating oxidative and inflammatory responses [20, 22]. Conversely, boron deficiency has been associated with reproductive toxicity, such as reduced sperm count, sperm abnormalities, and testicular atrophy in amphibians [23].

Multiple experimental and epidemiological studies have confirmed that boric acid is neither genotoxic nor mutagenic and may offer protection against several types of cancer, including prostate, breast, cervical, and lung cancers [14, 24]. In hepatocellular carcinoma (HCC) cell lines, boric acid has been shown to decrease cell viability in a dose- and time-dependent manner, inhibiting cell survival, colony formation, migration, and spheroid development. Populations consuming boron-rich diets or residing in regions with high boron levels in water and soil exhibit a reduced incidence of certain cancers [25, 26]. Further research indicates that boric acid mitigates the toxicity of various harmful agents, including heavy metals (e.g., vanadium [27], arsenic, bismuth, cadmium, mercury, and lead [28]), nanoparticles (e.g., titanium dioxide [29]), genotoxins (e.g., aflatoxin B1 [30], polycyclic hydrocarbons [3]), and anticancer drugs (e.g., paclitaxel [31]) by reducing chromosomal aberrations, micronucleus formation, and sister chromatid exchange. Additionally, dietary administration of boric acid, borax, colemanite, and ulexite in rats has enhanced antioxidant defenses and attenuated DNA damage induced by these genotoxins [32, 33]. However, literature also reports adverse effects of boric acid, particularly at higher concentrations. These include sterility in human peripheral lymphocytes [4], chromosomal and mitotic abnormalities in *Allium cepa* root tip cells [34], and DNA damage in zebrafish (*Danio rerio*) [19]. Cytotoxic effects, such as inhibition of cell proliferation and increased lipid peroxidation, have been observed across all tested concentrations (0.25–1%) [35]. Moreover, due to its antimicrobial activity at high doses, boric acid is employed as a bactericide, antiseptic, cleaning agent, and food preservative [36].

Human peripheral lymphocytes (HPLs) are widely utilized for assessing chromosomal and DNA damage, as they originate from pluripotent hematopoietic stem cells in the bone marrow and differentiate into B and T cells circulating between lymphoid organs and peripheral blood. Their abundance and accessibility make HPLs an ideal model for genotoxic and antigenotoxic evaluations [37, 38]. Moreover, DNA and chromosomal aberration frequencies in lymphocytes have been correlated with cancer risk and are elevated in cancer patients [39, 40].

Despite extensive research into the biological properties of boric acid, limited data exist regarding its simultaneous genotoxic and antigenotoxic effects using combined cytogenetic and DNA damage assays. While its protective effects against certain heavy metals and nanoparticles have been investigated, there is a lack of studies exploring its interaction with mitomycin C (MMC), an alkylating anticancer drug, in human lymphocytes. Thus, the present study aims to address this gap by evaluating the dual role of boric acid- as both a potential genotoxicant and a protective agent- against DNA and chromosomal damage induced by two mechanistically distinct genotoxicants: MMC and hydrogen peroxide (H₂O₂), the latter used as a positive control due to its ability to generate reactive oxygen species (ROS) and free radicals. To assess potential genotoxic and antigenotoxic effects, a battery of assays- Chromosomal Aberrations (CA), Sister Chromatid Exchange (SCE), Cytokinesis-Block Micronucleus Cytome (CBMN-cyt), and Comet assay- were employed using in vitro human peripheral lymphocytes.

## Materials and Methods

### Experimental Design

The study was conducted using human peripheral blood lymphocytes obtained from three healthy, non-smoking female donors aged between 22 and 30 years. All donors had no known history of medical conditions, had not taken any medications within the three months preceding the study, and reported no known exposure to genotoxic agents. Lymphocytes were isolated and cultured under standardized conditions for subsequent cytogenetic and DNA damage assays.

### Ethics of Experimentation

This research was approved by the Clinical Research Ethics Committee of the Gazi University Faculty of Medicine (Approval Date: 06 March 2023; Approval No: 10). All procedures adhered to the ethical principles outlined in the Declaration of Helsinki. Informed consent was obtained from all participants before their inclusion in the study.

### Chemicals

CAS and Cat numbers of chemicals and suppliers are summarized in Table 1.

**Table 1:** CAS/Cat numbers of chemicals and suppliers

CAS/Cat No	Chemical	Supplier
CY100-100	Chromosome medium LymphoPlus	Cegrogen Biotech
PBSH0500-540	Dulbecco's PBS	Cegrogen Biotech
J0100-840	Lymphocyte separation medium	Cegrogen Biotech
67–56-1	Methyl alcohol	Merck
64–19-7	Glacial acetic acid	Merck
1092040500	Giemsa	Merck
1079610100	Entellan	Merck
7447–40-7	Potassium chloride	Merck
50–00-0	Formaldehyde	Merck
1310–73-2	Sodium hydroxide	Merck
111374	Buffer tablets pH 6.8	Merck
59–14-3	Bromodeoxyuridine	Sigma Aldrich
64–86-8	Colchicine	Sigma Aldrich
14930–96-2	Cytochalasin-B	Sigma Aldrich
9002–93-1	Triton X-100	Applichem
6381–92-6	EDTA	Applichem
67–68-5	DMSO	Applichem
9012–36-6	Low melting agarose	Applichem
9012–36-6	Agarose low EEO	Applichem
77–86-1	Tris	Applichem
1239–48-8	Ethidium bromide	Applichem

### Test Materials

#### Boric Acid

**Fig. 1:**
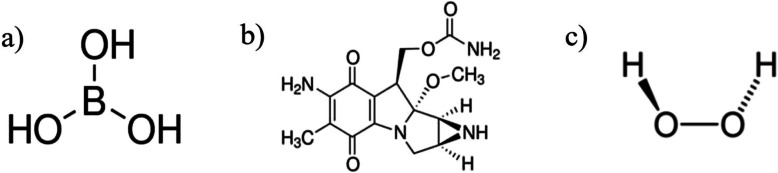
The structural formula of **a**) Boric acid (Eti Maden), **b**) Mitomycin C (Sigma-Aldrich), **c**) Hydrogen peroxide (Sigma-Aldrich)

Boric acid (Cas No: 10043–35-3) was from Eti Mine Works, Ankara, Türkiye. The chemical formula is H_3_BO_3_, and the molecular weight is 61,83 g/mol. Its structural formula is given in Fig. 1a.

#### Mitomycin C (MMC)

Mitomycin C, an antitumor antibiotic and alkylating agent, is isolated from *Streptomyces caespitosus*. In this study, MMC was commercially obtained from Sigma-Aldrich (CAS No: 50–07-7). Chemically, MMC: 6-amino-4,7-dioxo-8-methoxy-5-methyl-1,1,2,8,8,8-hexahydroazirin[2,3]pyrrolo[1,2]indole-8-methylcarbamate. It has a molecular formula of C₁₅H₁₈N₄O₅ and a molecular weight of 334.33 g/mol. The structural formula of MMC is presented in Fig. 1b.

#### Hydrogen Peroxide (H_2_O_2_)

Hydrogen peroxide (H₂O₂, 35%) was commercially obtained from Applichem (CAS No: 7722–84-1). As a potent oxidizing agent, H₂O₂ can react with organic and inorganic compounds, leading to significant oxidative damage to cellular components, including DNA. It has a molecular formula of H₂O₂ and a molecular weight of 34.01 g/mol. The structural formula of H₂O₂ is shown in Fig. 1c.

#### Determination of the Test Concentrations

A preliminary dose-range finding experiment was conducted to evaluate the cytotoxicity of boric acid (BA), following the OECD Test Guideline No. 473, which recommends that the highest test concentration should reduce the mitotic index (MI) to approximately 45 ± 5% of the concurrent negative control [41]. To determine suitable concentrations of BA for assessing its cytotoxic, genotoxic, and antigenotoxic potential in human lymphocytes exposed to DNA-damaging agents, relevant concentrations were selected based on a review of previous studies [27, 29, 33]. These studies commonly utilized BA concentrations of 2.5, 5, and 10 micromolar (µM) in one experimental set and 0.25, 0.5, 1, 2, 5, 10, and 20 parts per million (ppm) in another.

Building on this literature, a preliminary experiment was performed using 2.5, 5, 10, and 20 µg/mL of BA. The results indicated that 5, 10, and 20 µg/mL concentrations were substantially cytotoxic to lymphocytes. Consequently, lower concentrations- 0.25, 0.5, 1, and 2.5 µg/mL- were selected for use in the main experiments based on their minimal effects on the mitotic index.

According to standard protocols for evaluating antigenotoxic potential, experimental designs should include a negative control (untreated cells), a positive control (a known genotoxicant), and at least three different concentrations of the test compound [42]. Accordingly, four treatment concentrations of BA were included in this study. A stock solution of BA was prepared in distilled water and then diluted to the desired final concentrations for each treatment group.

#### Chromosome Aberrations and Sister Chromatid Exchange Assays

The chromosome aberration assay was performed in human peripheral lymphocytes using the method described by Evans et al. [43], with minor modifications outlined in subsequent studies [44]. The sister chromatid exchange assay was conducted following the protocol established by Perry and Wolf [45], with slight modifications [46, 47]. Heparinized whole blood (0.2 mL) collected from donors was added to culture tubes containing 2.5 mL of LymphoPlus medium supplemented with 5-bromo-2'-deoxyuridine (BrdU), a synthetic thymidine analog, at a final concentration of 10 µg/mL. BrdU incorporation during two successive rounds of cell division enabled the differential staining of sister chromatids [48]. Lymphocyte cultures were incubated at 37 °C for 72 h to allow for proliferation.

Four concentrations of boric acid (BA)−0.25, 0.5, 1, and 2.5 µg/mL- were administered to the HPL cultures. In addition, cultures were treated with 0.20 µg/mL of mitomycin C (MMC) as a positive control, as well as combinations of BA (at all four concentrations) with MMC (BA + MMC). Treatments were applied during the final 24 and 48 h of culture. The MMC concentration (0.20 µg/mL) was selected based on its widespread use in genotoxicity assays, where it induces detectable DNA damage without significant cytotoxicity, thereby serving as a reliable positive control [38, 47, 49]. A negative control group (treated with distilled water) was also included.

To arrest cells at the metaphase stage, 0.06 µg/mL of colchicine was added to each culture at the 70th hour of incubation. Following incubation, cultures were centrifuged, and cells were treated with a hypotonic solution (0.075 M KCl at 37 °C), then fixed three times using methanol: acetic acid (3:1). The fixed cell suspensions were dropped onto clean microscope slides and stained with 5% Giemsa (pH 6.8) for chromosomal analysis.

#### Cytogenetic Evaluation and Scoring Criteria

In each treatment and control group, 300 metaphase cells (100 per donor) were analyzed to determine the frequency of aberrant cells and chromosome aberrations per cell. Metaphases were selected and scored according to the following standardized criteria: (i) chromosome number within the range of 46 ± 2 centromeres; (ii) clear separation of chromosomes and arms with minimal overlap; (iii) distinguishable chromatids with intact centromeres; and (iv) well-defined, sharp chromosome morphology [49].

For the assessment of cytotoxicity, the mitotic index (MI) was calculated by evaluating 300 well-spread metaphases (100 per donor), corresponding to the percentage of mitotic cells among the total number of cells observed [48, 50]. MI served as a marker of cell proliferation and cytotoxicity, as recommended by OECD Test Guideline No. 473 [41].

The sister chromatid exchange was evaluated using a modified protocol based on Speit and Haupter [46]. Following cell fixation, slides were exposed to ultraviolet (UV) light at 254 nm (using a mercury lamp at a distance of 15 cm) for 13 min. Differential staining of sister chromatids was achieved with 5% Giemsa in Sorensen’s phosphate buffer (pH 6.8) for 5 min.

SCE frequency was determined by analyzing 75 well-spread metaphase cells (25 per donor) in the second mitotic division, each containing 46 ± 2 centromeres. SCEs were scored blindly by counting the number of dark-stained segments on light-stained chromatids or light-stained segments on dark-stained chromatids of each chromosome.

The replication index (RI), an indicator of proliferative activity, was calculated using the following formula: RI = [1 × (M1) + 2 × (M2) + 3 × (M3)]/N, where M1, M2, and M3 denote the number of cells in the first, second, and third mitotic divisions, respectively, and *N* represents the total number of cells evaluated [48].

#### Cytokinesis-Block Micronucleus Cytome Assay

The cytokinesis-block micronucleus cytome assay was performed according to the method described by Fenech [51], with minor modifications as previously reported [52]. Briefly, whole blood samples from donors were added to culture tubes containing 2.5 mL of LymphoPlus medium and incubated at 37 °C for 72 h. Boric acid was applied at 0.25, 0.5, 1, and 2.5 µg/mL concentrations, either alone or combined with mitomycin C (MMC; 0.20 µg/mL) (BA + MMC treatments) during the final 48 h of incubation. Cytochalasin B (5.2 µg/mL) was added at the 44th hour to inhibit cytokinesis and allow the accumulation of binucleated cells.

Distilled water and MMC (0.20 µg/mL) were negative and positive controls, respectively. At the end of the incubation period, cultures were treated with 0.075 M KCl for 5 min at 37 °C to induce hypotonic swelling. Cells were then fixed in methanol: acetic acid (3:1, v/v) through three consecutive fixation steps. The resulting cell suspension was dropped onto pre-cleaned, chilled microscope slides, which were subsequently air-dried and stained with 5% Giemsa at room temperature for approximately 10 min.

All slides were coded to ensure blinded scoring. Micronucleus evaluation was conducted according to the scoring criteria established by Fenech [51]. At least 3000 binucleated cells (1000 per donor) were analyzed for each treatment group to determine micronucleus frequency.

#### Assessment of Nuclear Division Index and Cytokinesis-Block Proliferation Index

To evaluate the proliferative activity and cytotoxicity of boric acid and BA + MMC combinations, 1500 cells (500 per donor) were analyzed for each treatment and control group. Two indices were calculated: the Nuclear Division Index (NDI) and the Cytokinesis-Block Proliferation Index (CBPI).

The CBPI was used as the primary indicator of cytotoxicity, following the guidelines of OECD Test Guideline 487. In parallel, NDI was calculated as an additional metric to assess cytostatic effects and lymphocyte mitogenic response, as recommended by Fenech [51].

The indices were computed using the following formulas [53]:

$$\mathrm{NDI}=\left(1\times\mathrm N1\right)+\left(2\times\mathrm N2\right)+\left(3\times\mathrm N3\right)+\left(4\times\mathrm N4\right)/\mathrm N\left(\mathrm N-\mathrm N4\;\mathrm{represent}\;\mathrm{the}\;\mathrm{number}\;\mathrm{of}\;\mathrm{cells}\;\mathrm{with}\;\mathrm{one}\;\mathrm{to}\;\mathrm{four}\;\mathrm{nuclei},\;\mathrm{respectively},\;\mathrm{and}\;\mathrm N\;\mathrm{is}\;\mathrm{the}\;\mathrm{total}\;\mathrm{number}\;\mathrm{of}\;\mathrm{cells}\;\mathrm{scored}\right).$$

$$\mathrm{CBPI}=\left[\left(1\times\mathrm{number}\;\mathrm{of}\;\mathrm{mononucleated}\;\mathrm{cells}\right)+\left(2\times\mathrm{number}\;\mathrm{of}\;\mathrm{binucleated}\;\mathrm{cells}\right)+\left(3\times\mathrm{number}\;\mathrm{of}\;\mathrm{multinucleated}\;\mathrm{cells}\right)+\left(3\;\mathrm{or}\;\mathrm{more}\right)\right]/\mathrm n,\;\mathrm{where}\;\mathrm n\;\mathrm{is}\;\mathrm{the}\;\mathrm{total}\;\mathrm{number}\;\mathrm{of}\;\mathrm{cells}\;\mathrm{evaluated}.$$

These parameters provide insight into the test compounds'cytotoxic and cytostatic potential on human peripheral lymphocytes.

### Alkaline Comet Assay

The alkaline comet assay was conducted to evaluate the DNA-damaging potential of boric acid and its protective effect against hydrogen peroxide (H₂O₂), following the protocol developed initially by Singh et al. [54], with minor modifications as previously reported [55]. This assay was used to assess DNA strand breaks induced by BA alone and in combination with H₂O₂ (BA + H₂O₂).

Human peripheral lymphocytes were isolated from whole blood using a lymphocyte separation medium. Cell viability, assessed by the trypan blue exclusion method, was ≥ 97%. Isolated lymphocytes were treated at 37 °C for 1 h with one of four concentrations of BA (0.25, 0.5, 1, and 2.5 µg/mL) or simultaneously with 100 µM H₂O₂ (BA + H₂O₂). Control groups included a negative control (distilled water) and a positive control (100 µM H₂O₂) [54].

Following treatment, the cells were centrifuged, resuspended in phosphate-buffered saline (PBS), and mixed with low-melting-point agarose. This cell-agarose suspension was pipetted onto microscope slides pre-coated with normal-melting-point agarose and maintained at 4 °C to solidify. Slides were then immersed in lysis solution (2.5 M NaCl, 100 mM EDTA, 10 mM Tris, 10% DMSO, and 1% Triton X-100, pH 10) for 1 h at 4 °C. DNA unwinding was performed in an electrophoresis buffer (1 mM EDTA, 300 mM NaOH, pH > 13), followed by electrophoresis at 25 V and 300 mA for 20 min.

Subsequently, slides were neutralized with 0.4 M Tris buffer (pH 7.5) and stained with ethidium bromide (20 µg/mL). For each treatment group, two slides per donor were prepared. Three hundred nucleoids (50 per slide, 100 per donor) were scored blindly using a fluorescent microscope (Olympus BX51) with a 546 nm excitation filter and a 590 nm barrier filter at 400 × magnification. DNA damage was quantified based on tail intensity (percentage of DNA in the tail) using a computer-assisted image analysis system (Comet Assay IV, Perceptive Instruments Ltd., UK).

### Statistical Analysis

All statistical analyses were performed using IBM SPSS Statistics version 23.0. One-way analysis of variance (ANOVA), followed by Tukey’s post hoc test, was employed to evaluate the dose-dependent effects of boric acid on various cytogenetic and DNA damage parameters. These included abnormal cell frequency, chromosome aberrations per cell (CA/Cell), sister chromatid exchanges per cell (SCE/Cell), mitotic index (MI), replication index (RI), micronucleus (MN) frequency, nuclear buds (NBUD), nucleoplasmic bridges (NPB), nuclear division index (NDI), cytokinesis-block proliferation index (CBPI), and comet assay tail intensity. All data are expressed as mean ± standard error of the mean (SEM), and statistical significance was considered at *p* < 0.05.

## Results

### Chromosome Aberrations Test

The results of the chromosome aberration test in human peripheral lymphocytes treated with Mitomycin C, boric acid, and their combinations (BA + MMC) are summarized in Table 2. Treatments were conducted for 24- and 48-h durations, with MMC applied at 0.20 µg/mL and BA at 0.5, 1, 2.5, and 5 µg/mL, either alone or in combination with MMC. As expected, MMC, serving as the positive control, significantly increased the frequency of aberrant cells and chromosome aberrations per cell (CA/Cell) at both exposure durations compared to the negative control (*p* < 0.05). This increase was approximately fivefold, reflecting strong genotoxic potential in human lymphocytes. MMC induced a variety of chromosomal aberrations, including seven structural types-chromatid breaks (most frequent), chromosome breaks, fragments, sister chromatid unions, dicentric chromosomes, chromatid exchanges, and ring chromosomes-as well as one numerical aberration (polyploidy), although the latter appeared infrequently. In contrast, at any concentration tested, BA alone did not significantly elevate the CAs or CA/Cell frequency relative to the negative control, suggesting no genotoxic effect under the tested conditions. However, a non-significant upward trend was observed with increasing concentrations.

**Table 2:** Effect of Boric acid (BA) and combined BA + MMC treatment on chromosome aberrations

Test substance	Treatment		Aberrations									Abnormal cell ± SEM (%)	Protective effect (%)	CA/Cell ± SEM	Protective effect (%)
	Time (h)	Conc. (μg/mL)	ctb	csb	f	dc	scu	cte	r	p	e				
NC	24	0.00	6	1	1	-	1	-	-	-	-	3.00 ± 0.58		3.00 ± 0.58	
PC	24	0.20	28	6	5	2	5	-	-	3	-	15.00 ± 1.00^**a**^		16.33 ± 1.76^**a**^	
BA	24	0.25	11	1	5	1	2	-	1	2	-	6.33 ± 1.45		7.67 ± 2.33	
		0.5	7	1	3	-	5	-	-	2	-	5.33 ± 0.88		6.00 ± 1.15	
		1	11	2	-	-	1	1	-	7	-	7.33 ± 1.20		7.33 ± 1.20	
		2.5	12	2	2	-	2	-	-	3	-	6.67 ± 0.67		7.00 ± 0.58	
BA + MMC	24	0.25	12	3	1	1	-	1	-	3	-	6.67 ± 1.86*	69.41	7.00 ± 2.00*	69.99
		0.5	3	1	4	1	3	-	-	5	1	6.00 ± 0.58*	75.00	6.00 ± 0.58*	77.49
		1	16	3	2	1	1	-	-	1	-	8.33 ± 0.88*	55.58	7.33 ± 1.45*	67.51
		2.5	18	3	1	-	2	-	-	1	-	8.00 ± 2.08*	58.33	8.33 ± 2.40	60.01
NC	48	0.00	5	2	1	-	-	-	-	3	-	3.67 ± 0.33		3.67 ± 0.33	
PC	48	0.20	28	8	7	2	2	2	1	2	-	16.33 ± 1.45^**a**^		17.33 ± 2.03^**a**^	
BA	48	0.25	11	1	1	-	5	-	-	1	-	5.33 ± 0.33		6.33 ± 0.88	
		0.5	9	1	1	1	1	-	-	3	-	5.33 ± 0.88		5.33 ± 0.88	
		1	7	5	2	1	3	-	-	1	-	5.67 ± 0.33		6.33 ± 0.88	
		2,5	13	2	1	-	3	-	-	-	-	6.33 ± 1.20		6.33 ± 1.20	
BA + MMC	48	0.25	8	2	1	-	1	-	-	1	-	4.33 ± 0.88*	94.71	4.33 ± 0.88*	95.16
		0.5	8	4	1	-	2	-	-	1	-	5.33 ± 0.33*	86.88	5.33 ± 0.33*	87.84
		1	12	3	4	-	3	-	1	-	-	7.00 ± 1.15*	73.69	7.67 ± 0.67*	70.71
		2.5	14	5	3	-	2	-	-	1	-	7.67 ± 0.33*	68.40	8.33 ± 0.88*	65.88

*NC* Negative Control, *PC* Positive Control-MMC-Mitomycin-C, *Conc.* Concentration, *SEM* Standard error of the mean, *ctb* chromatid break, *csb* chromosome break, *f* fragment, *dc* dicentric chromosome, *scu* sister chromatid union, *cte* chromatid exchange, r: ring chromosome, p: polyploidy, e: endoreduplication

^a^Significantly different from the negative control, *p*<0.05 (One-Way ANOVA test); *Significantly different from the positive control, *p*<0.05 (One-Way ANOVA test)

Notably, concurrent treatment with BA and MMC resulted in a significant reduction (*p* < 0.05) in both the frequency of aberrant cells and CA/Cell across all BA concentrations (except for CA/Cell at 2.5 µg/mL, 24 h) when compared to MMC alone. Although these protective effects were not strictly dose-dependent, the most pronounced reductions were observed at the two lowest BA concentrations (0.25 and 0.5 µg/mL). At 24 h, 0.5 µg/mL BA reduced aberrant cell frequency and CA/Cell by 75% and 77.47%, respectively, while at 48 h, the reductions reached 94.71% and 95.16% at 0.25 µg/mL. Overall, BA exhibited a greater ameliorative effect at 48 h than 24 h, suggesting enhanced protective efficacy with prolonged exposure. Importantly, while BA significantly mitigated MMC-induced chromosomal damage, it did not fully restore CA frequencies to baseline (control) levels. These findings support the potential antigenotoxic role of boric acid in counteracting the clastogenic effects of MMC without exhibiting inherent genotoxicity.

### Sister Chromatid Exchange Test

**Fig. 2:**
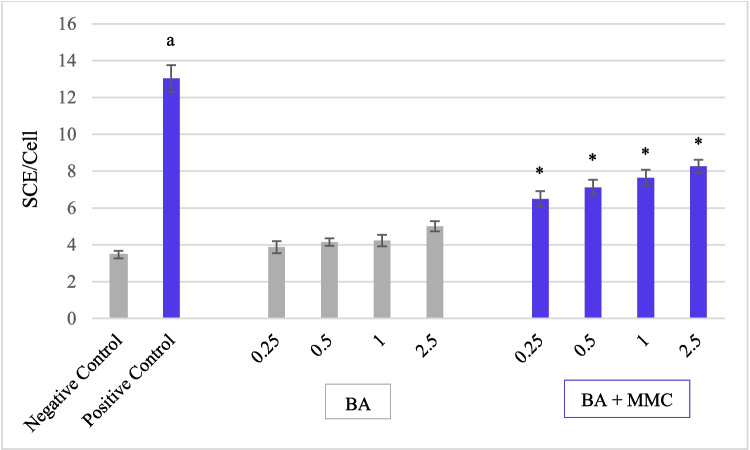
Effect of Boric acid (BA) and combined BA + MMC treatment on sister chromatid exchange frequency per cell (SCE/Cell) after a 24-h exposure in human peripheral lymphocytes. Data are presented as mean ± standard error of the mean (SEM) from three independent donors (*n* = 3). Statistical analysis was performed using one-way ANOVA followed by Tukey's post hoc test."a"indicates a significant difference compared to the negative control (*p* < 0.05),"*"indicates a significant difference compared to the positive control (*p* < 0.05)

**Fig. 3:**
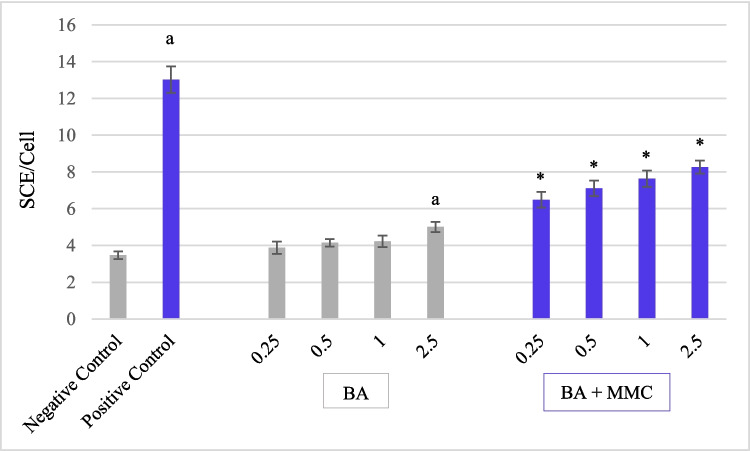
Effect of Boric acid (BA) and combined BA + MMC treatment on sister chromatid exchange frequency per cell (SCE/Cell) after a 48-h exposure in human peripheral lymphocytes. Data are presented as mean ± standard error of the mean (SEM) from three independent donors (*n* = 3). Statistical analysis was performed using one-way ANOVA followed by Tukey's post hoc test."a"indicates a significant difference compared to the negative control (*p* < 0.05),"*"indicates a significant difference compared to the positive control (*p* < 0.05)

The effects of mitomycin C, boric acid, and their combined treatments (BA + MMC) on sister chromatid exchange, replication index, and mitotic index are summarized in Table 3 and illustrated in Figs. 2 and 3. Treatments were applied to human peripheral lymphocytes for 24 and 48 h to evaluate both genotoxic and antigenotoxic potentials. Compared to the negative control, exposure to MMC alone significantly increased the number of SCEs per cell (*p* < 0.05) at both time points. SCEs per cell ranged from 2 to 18 at 24 h and 4 to 31 at 48 h, demonstrating a time-dependent increase in MMC-induced genotoxicity. In contrast, none of the tested concentrations of BA alone (except for 2.5 µg/mL at 48 h) significantly elevated SCE frequency relative to the negative control, indicating no substantial genotoxic effect under these conditions. Co-treatment with BA and MMC at all four BA concentrations (0.25, 0.5, 1, and 2.5 µg/mL) significantly reduced MMC-induced SCEs per cell (*p* < 0.05) at both time points, although this protective effect was not dose-dependent. The most effective reduction occurred at the lowest BA concentration (0.25 µg/mL), with SCE frequency reductions of 70.19% at 24 h and 68.41% at 48 h. Other concentrations also showed protective effects, with the following cuts in SCEs: At 24 h: 0.5 µg/mL: 66.76%, 1 µg/mL: 64.97%, 2.5 µg/mL: 59.16%. At 48 h: 0.5 µg/mL: 61.91%, 1 µg/mL: 56.38%, 2.5 µg/mL: 49.79%. These results indicate that BA confers a significant protective effect against MMC-induced sister chromatid exchanges in human lymphocytes, with a more pronounced impact observed at lower concentrations. However, BA could not fully reverse the genotoxic damage caused by MMC to control levels.

**Table 3:** Effect of Boric acid (BA) and combined BA + MMC treatment on SCE, RI, and MI

Test substance	Treatment			M_1_	M_2_	M_3_	RI ± SEM	MI ± SEM
	Time (h)	Conc (µg/mL)	Min-maks SCE					
NC	24	0.00	0–9	56	75	169	2.38 ± 0.88	10.27 ± 0.12
PC	24	0.20	2–18	47	86	167	2.40 ± 0.88	6.60 ± 1.06^a^
BA	24	0.25	1–10	45	79	176	2.44 ± 0.89	9.5 ± 0.23
		0.5	1–7	50	76	174	2.41 ± 0.88	9.37 ± 0.91
		1	1–11	41	79	180	2.46 ± 0.89	9.27 ± 0.03
		2.5	2–11	53	78	169	2.39 ± 0.88	7.23 ± 1.17
BA + MMC	24	0.25	0–11	37	81	182	2.48 ± 0.90	8.03 ± 0.69
		0.5	1–13	46	80	174	2.43 ± 0.88	8.33 ± 0.24
		1	1–13	54	75	171	2.39 ± 0.88	8.57 ± 0.47
		2.5	1–14	49	86	165	2.39 ± 0.88	6.97 ± 1.03
NC	48	0.00	1–10	57	78	165	2.36 ± 0.88	13.27 ± 1.52
PC	48	0.20	4–31	59	83	158	2.33 ± 0.87	8.00 ± 0.45^a^
BA	48	0.25	0–17	54	75	171	2.39 ± 0.88	10.20 ± 1.17
		0.5	1–7	67	78	155	2.29 ± 0.86	9.27 ± 0.57
		1	0–12	45	80	175	2.43 ± 0.89	9.17 ± 0.62
		2.5	1–12	47	83	170	2.46 ± 0.89	9.43 ± 0.92
BA + MMC	48	0.25	0–17	44	79	177	2.44 ± 0.89	8.53 ± 0.38
		0.5	2–16	39	88	173	2.45 ± 0.89	8.57 ± 0.12
		1	1–16	48	83	169	2.40 ± 0.88	8.27 ± 0.89
		2.5	1–20	44	90	166	2.41 ± 0.88	8.07 ± 0.33

*NC* Negative Control, *PC* Positive Control-MMC-Mitomycin-C, *Conc.* Concentration, *SEM* Standard error of the mean, *SCE* sister chromatid exchange, *M1* mitosis 1, *M2* mitosis 2, *M3* mitosis 3, *RI* replication index, *MI* mitotic index

^a^Significantly different from the negative control, *p* < 0.05 (One-Way ANOVA test)

In summary, the SCE findings are consistent with the outcomes observed in the chromosome aberration test, reinforcing the conclusion that boric acid does not exhibit intrinsic genotoxicity and possesses a notable antigenotoxic capacity against MMC-induced chromosomal damage.

In this study, neither the positive control nor any tested concentration of boric acid caused a statistically significant alteration in the replication index at either treatment interval compared to the negative control (Table 3). Likewise, co-administration of boric acid and mitomycin C did not significantly affect the RI at either 24 or 48 h compared to the positive control. Conversely, MMC treatment alone resulted in a marked and statistically significant reduction in the mitotic index at both time points compared to the negative control. While individual administration of varying concentrations of BA did not significantly affect the MI, co-treatment with BA and MMC led to modest but non-significant elevations in MI across all BA concentrations when compared to MMC alone. Nevertheless, BA co-treatment did not restore the MI to baseline levels observed in the untreated control group.

### Cytokinesis-Block Micronucleus Cytome Test

This study also examined the effects of individual treatments with boric acid and mitomycin C, as well as their combined administration (BA + MMC), on the formation of micronuclei, nucleoplasmic bridges, nuclear buds, the cytokinesis-block proliferation index, and the nuclear division index following 48 h of exposure. The results are summarized in Table 4. MMC treatment alone resulted in a statistically significant increase in MN frequency compared to the negative control (*p* < 0.05). In contrast, none of the concentrations of BA administered alone produced a substantial change in MN frequency. Moreover, co-treatment with BA and MMC significantly reduced MN frequency compared to MMC treatment alone (*p* < 0.05) at all concentrations tested. However, this reduction did not follow a dose-dependent pattern. The two lowest concentrations of BA demonstrated the most significant and identical protective effect, each reducing MN formation by 77.24%. The remaining two concentrations provided 72.75% and 68.11% protection, respectively. Although BA could not fully restore MN frequency to levels observed in the negative control group, these findings suggest that BA exerts a chemopreventive effect against MMC-induced genotoxicity in lymphocytes.

**Table 4:** Effect of Boric acid (BA) and combined BA + MMC treatment on MN, NBUD, NPB, NDI, and CBPI

Test substance	Treatment		Num. BN cells scored	MN frequencies in BN cells			MN (‰) ± SEM	Protective effect (%)	NBUD ± SEM	NPB ± SEM	NDI ± SEM	CBPI ± SEM
	Time (h)	Conc. (µg/mL)		1	2	3						
NC	48	0.00	3000	7	-	-	2.33 ± 1.20	-	1.67 ± 0.33	0.67 ± 0.33	2.01 ± 0.36	1.92 ± 0.36
PC	48	0.20	3000	27	2	-	9.67 ± 0.88^**a**^	-	6.67 ± 1.8^**a**^	2.67 ± 1.76	1.94 ± 0.36	1.86 ± 0.34
BA	48	0.25	3000	9	-	-	3.00 ± 1.00	-	2.00 ± 1.15	1.00 ± 1.00	1.85 ± 0.35	1.93 ± 0.36
		0.5	3000	8	2	-	3.33 ± 0.33	-	1.67 ± 0.33	0.67 ± 0.33	2.09 ± 0.37	2.00 ± 0.36
		1	3000	10	-	-	3.33 ± 0.33	-	2.00 ± 1.00	1.00 ± 0.58	1.90 ± 0.35	1.83 ± 0.34
		2.5	3000	11	-	-	3.67 ± 0.33	-	2.33 ± 0.33	0.67 ± 0.33	1.82 ± 0.35	1.75 ± 0.33
BA + MMC	48	0.25	3000	11	-	1	4.00 ± 1.00*	77.24	3.00 ± 1.53	1.00 ± 0.58	1.76 ± 0.35	1.70 ± 0.33
		0.5	3000	12	-	-	4.00 ± 1.15*	77.24	2.67 ± 0.88	0.00 ± 0.00	1.90 ± 0.35	1.83 ± 0.34
		1	3000	13	-	-	4.33 ± 0.88*	72.75	2.67 ± 0.33	1.00 ± 0.58	1.86 ± 0.35	1.79 ± 0.34
		2.5	3000	14	-	-	4.67 ± 0.33*	68.11	2.67 ± 0.33	1.00 ± 0.00	1.67 ± 0.33	1.62 ± 0.33

*NC* Negative Control, *PC* Positive Control-Mitomycin-C (MMC), *Conc.* Concentration, Num. BN cells: Number of binucleate cells, *SEM* standard error of the mean. *MN* micronucleus, *BN* binucleate, *NBUD* nuclear bud, *NPB* nucleoplasmic bridge, *NDI* nuclear division index, *CBPI* cytokinesis-block proliferation index

^a^Significant compared to the negative control, *p* < 0.05 (One-Way ANOVA test); *Significant compared to the positive control, *p* < 0.05 (One-Way ANOVA test)

Mitomycin-C significantly increased nuclear bud frequency compared to the control. BA alone did not considerably affect NBUD formation, and co-treatment with BA and MMC led to a non-significant reduction in NBUD frequency compared to MMC alone. Similarly, MMC treatment resulted in an approximately fourfold, though non-significant, increase in nucleoplasmic bridge frequency. Neither BA alone nor the combined BA + MMC treatment produced statistically significant changes in NPB frequency compared to the negative and positive control. Furthermore, neither MMC nor its combination with BA had any considerable effect on NDI or CBPI, indicating no substantial impact on cell proliferation or nuclear division dynamics under the tested conditions.

### Comet Test

**Fig. 4:**
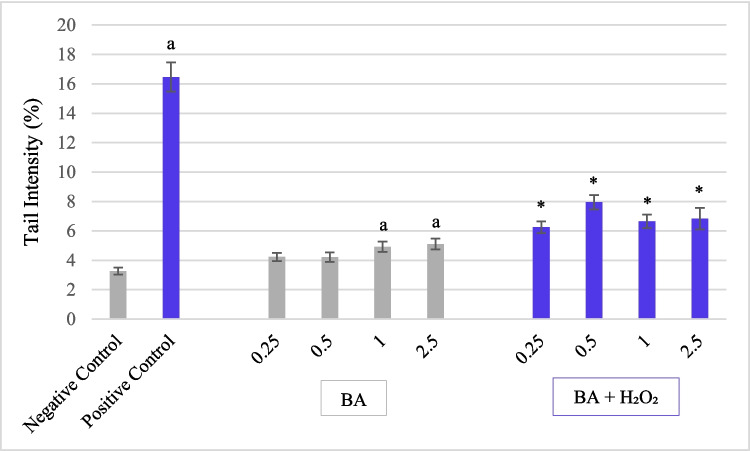
Effect of Boric acid (BA) and combined BA + MMC treatment in tail intensity (%) after one-hour exposure in human peripheral lymphocytes. Data are presented as mean ± standard error of the mean (SEM) from three independent donors (*n* = 3). Statistical analysis was performed using one-way ANOVA followed by Tukey's post hoc test."a"indicates a significant difference compared to the negative control (*p* < 0.05);"*"indicates a significant difference compared to the positive control (H₂O₂) (*p* < 0.05)

**Fig. 5:**
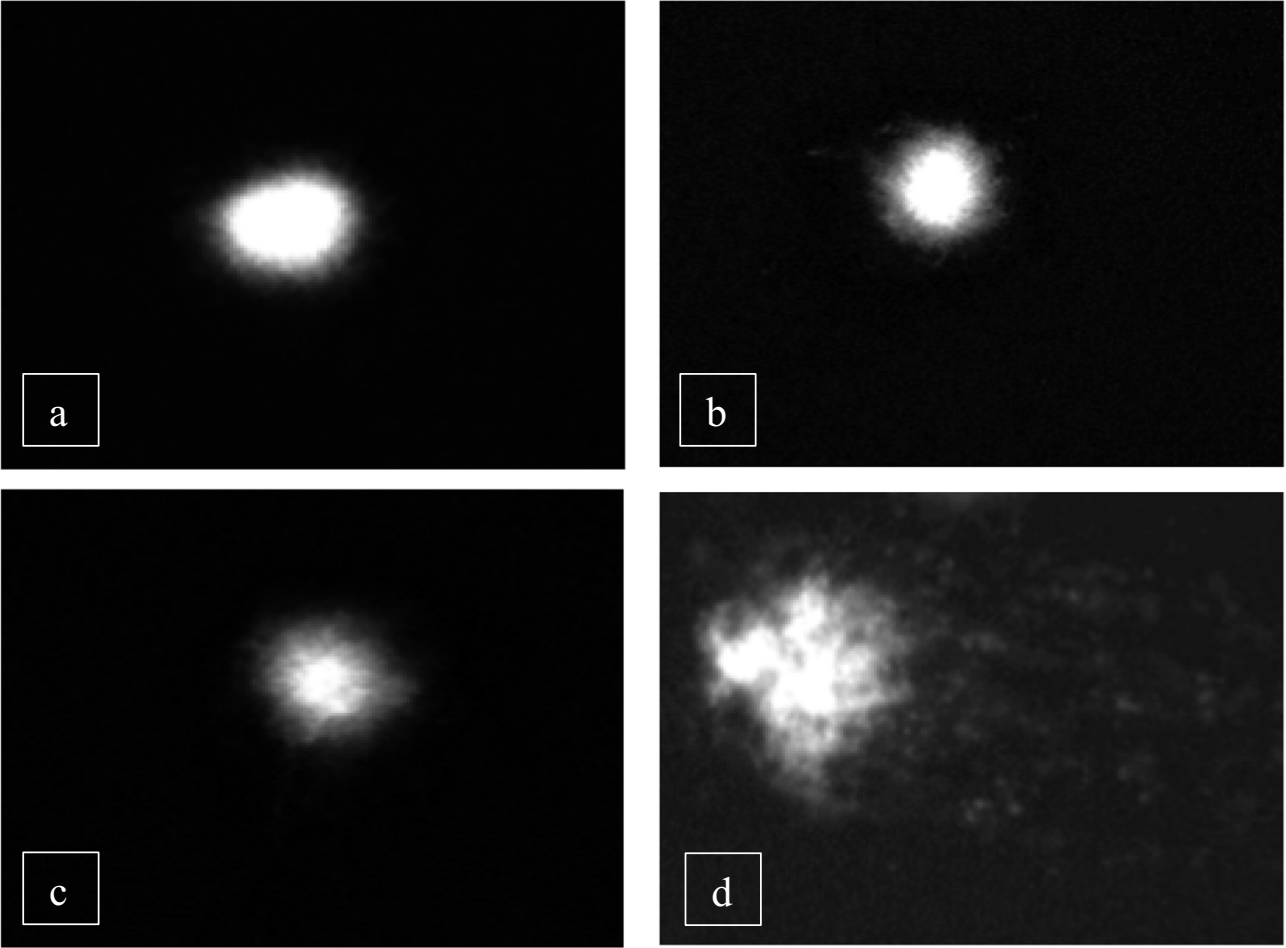
Images from a comet assay showing different levels of DNA damage (tail intensity) in human peripheral lymphocytes treated with Boric acid (BA) and BA + H₂O₂ combination for one hour. **a**) Undamaged DNA, **b**) Slightly damaged DNA, **c**) Moderately damaged DNA, **d**) Highly damaged DNA

In this study, hydrogen peroxide, employed as the positive control, significantly increased comet tail intensity (expressed as % DNA in tail) in isolated human lymphocytes following a one-hour exposure, compared to the negative control (*p* < 0.05). On the other hand, single exposures to boric acid at 1 and 2.5 µg/mL concentrations resulted in a statistically significant elevation in tail intensity relative to the negative control (*p* < 0.05) but not at the lower concentrations. However, co-treatment with BA and H₂O₂ at all tested concentrations of BA significantly attenuated H₂O₂-induced DNA damage, as evidenced by reduced tail intensity (p < 0.05). The most pronounced protective effect was observed in the group co-treated with 0.25 µg/mL BA and H₂O₂, indicating a potential genoprotective role of BA against oxidative DNA damage (Figs. 4–5).

## Discussion

Boron, predominantly found in boric acid and borate minerals, plays essential biological roles because it can form complexes with biomolecules at physiological pH [13]. Numerous studies have demonstrated that boron-containing compounds can influence bone metabolism, hormonal activity, cognitive function, and the cellular response to oxidative stress [13, 32]. Boric acid, one of the boron compounds, has been widely employed across various sectors-including medicine, food preservation, and agriculture- due to its catalytic, antimicrobial, antioxidant, and anticancer properties [2, 10, 16, 31]. Additionally, boric acid has been reported to possess antigenotoxic and chemopreventive effects, protecting against heavy metal toxicity and other genotoxic agents [3, 14, 24, 27, 28]. Nonetheless, some reports have highlighted the potentially toxic effects of boric acid following prolonged or excessive exposure [19, 36, 56, 57].

In light of these dual effects, the present study aimed to assess both the genotoxic potential of boric acid and its protective efficacy against mitomycin C and hydrogen peroxide in vitro, using human peripheral blood lymphocytes. A battery of cytogenetic and DNA damage assays- including chromosome aberration, sister chromatid exchange, cytokinesis-block micronucleus cytome, and comet assays- was employed to evaluate chromosomal and primary DNA damage comprehensively. These assays are widely recognized for their utility in detecting both genotoxic and cytotoxic effects and assessing the antigenotoxic potential of test compounds against established mutagenic agents [47].

In the chromosome aberration assay, MMC significantly increased the frequency of aberrant cells and the mean number of aberrations per cell at 24- and 48-h exposure periods, compared to the negative control. In contrast, boric acid alone did not produce significant genotoxic effects at either time. Moreover, co-treatment with BA and MMC significantly reduced MMC-induced chromosomal aberrations and aberrations per cell, suggesting a protective- and potentially antigenotoxic- role for BA against MMC-induced clastogenicity. The most pronounced protective effect was observed at the lowest BA concentration following 48-h exposure, with reduced chromosomal damage exceeding 95%. MMC functions as a bifunctional alkylating agent, capable of forming DNA-DNA or DNA–protein crosslinks, thereby blocking transcription and replication by preventing DNA strand separation. Through enzymatic bioreduction, MMC is converted into a highly reactive intermediate, mitosene, which contains electrophilic sites capable of alkylating cellular nucleophiles such as DNA and glutathione (GSH). This process disrupts redox homeostasis and induces oxidative stress [58–60]. The resultant chromosomal aberrations, oxidative damage, and genomic instability are considered hallmark cytotoxic lesions, contributing to the inhibition of cell proliferation and apoptosis in rapidly dividing cancer cells. While this mechanism underlies MMC's therapeutic efficacy in cancer treatment, it also poses a risk to normal, proliferating cells. Inadequately repaired damage may result in persistent genomic instability, thereby increasing the likelihood of secondary mutations and chromosomal aberrations, possibly contributing to developing secondary malignancies. In addition to therapeutic exposures, humans are continually exposed to various environmental genotoxicants capable of inducing similar genomic instabilities. MMC has been extensively documented to cause chromosomal aberrations, SCEs, micronucleus formation, and DNA strand breaks in human lymphocytes [45, 49]. Accordingly, it is recommended as a positive control in genotoxicity testing protocols established by the Organisation for Economic Co-operation and Development (OECD Test No. 473; Test No. 487) [41, 61] and is routinely used in scientific research [16, 27, 47, 62]. Chromosomal aberrations observed during metaphase may arise through several mechanisms, including (i) double-strand breaks caused by replication errors or genotoxic agents, (ii) structural DNA damage from agents that intercalate or covalently bind DNA, and (iii) DNA crosslinking coupled with inhibition of DNA synthesis [63]. Genomic instability and chromosomal abnormalities, whether structural or numerical, are implicated in a broad range of human diseases, including stem cell dysfunction, metabolic disorders, infertility, chronic inflammation, and neurodegenerative conditions such as Alzheimer's and Parkinson's disease. Furthermore, these genomic alterations are closely associated with various cancers' initiation, progression, and metastasis [50, 64].

Protecting healthy cells from genotoxic damage is critical to minimizing long-term biological consequences, including genomic instability and carcinogenesis. As such, significant research efforts have been directed toward identifying genoprotective or antigenotoxic agents capable of counteracting the deleterious effects of genotoxins. Antigenotoxic compounds exert their protective effects through various mechanisms, most notably by acting as antioxidants that neutralize free radicals and reactive oxygen species (ROS) generated by genotoxic agents. Antioxidants can be either endogenous or exogenous and are commonly found in plant extracts, foods, and dietary boron intake. In addition to their antioxidant properties, antigenotoxic agents may enhance cellular DNA repair mechanisms, facilitating the accurate repair of genetic lesions and thereby preserving genomic integrity [16, 65].

Boric acid, a compound of considerable interest due to its low cost and broad biological activity, has demonstrated protective effects against genotoxic stress. In the present study, BA significantly reduced the frequency of aberrant cells and chromosomal aberrations per cell induced by mitomycin C, indicating its antigenotoxic potential. These findings suggest that BA may play a meaningful role in mitigating the harmful side effects of genotoxins such as MMC, an anticancer agent known for its genotoxicity in healthy dividing cells. Notably, this study is the first to report the protective effect of BA against MMC-induced chromosomal aberrations in human peripheral blood lymphocytes. Nevertheless, the observed protective effect of BA is consistent with previous studies that have reported similar antigenotoxic activities against various genotoxic chemicals in different cell types, cell lines, and organisms. For instance, Türkez et al. [4] assessed the genotoxicity of several borons, including borax, boric acid, colemanite, and ulexite at concentrations of 15, 50, and 300 mg/L using the CA test in human PBLs. Their results indicated that none of these boron compounds induced significant genotoxic effects. Furthermore, borax and boric acid significantly reduced vanadium-induced chromosomal aberrations, likely through attenuation of vanadium's suppressive effect on total antioxidant capacity [27]. Potassium tetraborate (PTB), synthesized from boric acid, potassium hydroxide, and water, has applications in detergents, disinfectants, and ophthalmic solutions. PTB did not demonstrate genotoxicity at concentrations ranging from 1.25 to 1280 µg/mL and significantly increased total antioxidant capacity (TAC) at lower concentrations (1.25–5 µg/mL) without affecting total oxidative status (TOS) [66].

Several boron-based therapeutic agents-such as borinic picolinate (AN0128), benzoxaborole derivatives (AN2728, AN2898), and the boron-containing protein synthesis inhibitor AN3365-have shown no evidence of genotoxic or mutagenic effects. Notably, tavaborole (AN2690), an antifungal agent, demonstrated no carcinogenic potential in long-term (two-year) animal studies. These findings support the premise that boron-containing therapeutics can be developed with minimal genetic toxicity risk [10]. In a related study, Arslan et al. [3] reported that boric acid, borax, and ulexite did not induce genotoxicity in human peripheral blood mononuclear cells. Instead, these compounds conferred chemopreventive effects by counteracting oxidative damage and reducing levels of malondialdehyde (MDA), 8-hydroxy-2'-deoxyguanosine (8-OH-dG), and chromosomal aberrations induced by 2,3,7,8-tetrachlorodibenzo-p-dioxin (TCDD), a potent environmental toxin. Likewise, other studies have demonstrated that boric acid and borax mitigate genotoxic effects induced by 3-chloro-1,2-propanediol through upregulation of antioxidant enzymes and reductions in ROS and intracellular calcium levels [16, 28, 67]. It has been proposed that boron reduces oxidative stress by modulating cellular redox systems, for example, by decreasing NADPH oxidase activity and γ-glutamyl transpeptidase activity, thereby increasing intracellular glutathione (GSH) levels, a critical antioxidant [21]. Boric acid has also been shown to enhance the activity of superoxide dismutase (SOD), which converts superoxide radicals to hydrogen peroxide, and catalase (CAT), which further detoxifies hydrogen peroxide into water and oxygen, thereby alleviating oxidative stress and preventing DNA damage [10].

Despite these beneficial effects, some studies have raised concerns regarding BA’s potential toxicity at high doses. Arslan et al. [56] reported that BA concentrations ranging from 400 to 1000 µg/mL significantly increased structural chromosomal aberrations in human PBLs after 24 and 48 h of exposure, likely by disrupting the DNA phosphodiester backbone. In vivo studies in Swiss Albino mice also indicated that prolonged exposure to high doses of BA (above 115 mg/kg for more than six weeks) induced oxidative stress in testicular tissue and negatively impacted sperm quality and DNA integrity [68]. At concentrations between 0.25% and 1.00%, BA exhibited pro-oxidant activity, as reflected by increased levels of MDA and elevated activities of antioxidant enzymes such as SOD and glutathione S-transferases (GSTs), suggesting a compensatory enzymatic response to oxidative stress [35]. The variability in reported outcomes may stem from differences in experimental conditions, including cell type, exposure duration, concentration, and assay methodology. These findings underscore the importance of careful dose selection and context-specific evaluation when considering boric acid as a chemopreventive agent.

Sister chromatid exchange is a sensitive cytogenetic assay that visually detects the reciprocal exchange of DNA segments between sister chromatids, serving as a biomarker of exposure to genotoxic agents [48, 56]. SCEs represent an early indicator of chromosomal instability and are typically elevated following exposure to clastogenic or mutagenic compounds. Although the in vitro SCE assay in mammalian cells was removed from the OECD test guidelines in 2014, it remains a valuable tool in genotoxicity assessment due to its high sensitivity and ability to detect subtle DNA damage. Furthermore, it provides insight into the antigenotoxic potential of compounds when a reduction in SCE frequency is observed compared to a positive control. Notably, SCE assays can detect low-dose genotoxic or antigenotoxic effects that may not induce overt structural chromosomal abnormalities [63]. In the present study, MMC significantly elevated the frequency of SCEs per cell in human peripheral blood lymphocytes, consistent with its known mechanism as a DNA crosslinking agent. In contrast, boric acid did not induce a statistically significant increase in SCE frequency at any tested concentration, except the highest dose (2.5 µg/mL), following 24- and 48-h exposures. More importantly, co-treatment with BA significantly attenuated MMC-induced SCE frequency across all concentrations tested. BA concentrations demonstrated up to 50% protection, indicating a pronounced ameliorative effect against MMC-induced genotoxicity. SCEs are primarily the result of DNA double-strand breaks (DSBs), one of the most lethal forms of genotoxic damage. These breaks are typically repaired by homologous recombination (HR) or non-homologous end joining (NHEJ). The HR repair pathway, in particular, utilizes the sister chromatid as a template, which results in SCE formation [50]. Many genotoxic agents that stall DNA replication forks or induce replication stress -such as MMC- can trigger SCEs by generating single-strand breaks that convert to DSBs during the S phase. HR-mediated fork restart upon encountering such lesions is believed to be a key mechanism underlying SCE induction [69].

Several proteins involved in DNA repair pathways influence SCE frequency. XRCC1 (X-ray repair cross-complementing protein 1) plays a crucial regulatory role in base excision repair (BER) and single-strand break repair. XRCC1 deficiency is associated with significantly elevated SCE levels. Similarly, dysfunction of other key proteins, such as PARP-1 and DNA ligase III, leads to increased SCEs, likely due to impaired repair of single-strand breaks, resulting in replication-associated DSBs and subsequent HR-mediated repair [48, 70]. These findings underscore the broader relationship between impaired DNA repair mechanisms and increased genomic instability. In healthy individuals, the baseline frequency of endogenous SCEs in PBLs typically ranges from 6 to 8 exchanges per cell [71]. However, significantly elevated SCE frequencies have been reported in the PBLs of patients with various malignancies-including breast, prostate, gastric, ovarian, and cervical cancers-where values may rise to threefold higher than the normal range [72]. In individuals with Bloom's syndrome, a rare autosomal recessive disorder characterized by genomic instability and cancer predisposition, SCE frequency averages around 89 exchanges per diploid cell. Despite these associations, subsequent research has produced inconsistent findings regarding the utility of SCE frequency as a biomarker for cancer risk [73]. Nonetheless, the potential for chemotherapy-induced DNA damage to promote secondary malignancies remains a significant clinical concern. Interindividual variation in DNA repair capacity also influences treatment response and toxicity profiles. Therefore, the SCE assay could be a predictive biomarker for evaluating individual sensitivity to anticancer drugs. As demonstrated in the current study, boric acid, particularly at its lowest tested concentration, was most effective in reducing MMC-induced SCEs. These findings suggest that low concentrations of BA may help mitigate the genotoxic side effects of chemotherapeutic agents such as MMC. In conclusion, the adjunctive use of boric acid alongside antineoplastic therapies may offer a promising strategy to reduce off-target DNA damage and improve the safety profile of cancer treatments [69].

Our findings regarding the sister chromatid exchange assay are consistent with prior studies evaluating boron compounds' genotoxic and antigenotoxic properties. For instance, Arslan et al. [56] assessed the genotoxic potential of boric acid (400–1000 µg/mL) in human peripheral blood lymphocytes using the SCE assay. They reported no significant changes in SCE frequency at either 24 or 48 h, except at the highest concentration (1000 µg/mL) after 48 h, resulting in insufficient analyzable cells due to excessive cytotoxicity. Similarly, Türkez et al. [4] demonstrated that exposure to boric acid, borax, colemanite, and ulexite did not increase the number of SCEs per cell compared to the negative control. Although these boron compounds did not induce genotoxic effects, they significantly modulated antioxidant enzyme activity in human PBLs. However, they also noted that exposure to 400 mg/L of borax and colemanite and 500 mg/L of all four boron compounds led to sterility in PBL cultures, indicating a threshold beyond which toxicity might arise. In another study, Geyikoglu and Türkez [27] found no genotoxic effects of borax and boric acid based on SCE frequencies. Instead, both compounds significantly reduced vanadium tetraoxide-induced SCEs, likely due to their ability to form diester bridges with cis-hydroxyl-containing molecules, a structural feature contributing to their antioxidant and protective effects. Additionally, borax and boric acid markedly attenuated titanium dioxide-induced SCEs by enhancing the antioxidant defense system [29]. Boric acid at concentrations of 2.5 and 5 mg/L has also been shown to be non-genotoxic based on SCE frequencies and to exert a protective effect against paclitaxel-induced SCEs by mitigating free radical generation [31]. Similarly, borax demonstrated chemoprotective effects by reducing the elevated SCE frequencies induced by aflatoxin B1, likely through attenuation of pro-oxidant activity [30]. However, contrary findings have also been reported. Pongsavee [57] observed that all tested concentrations of borax (0.15, 0.2, 0.3, and 0.6 mg/mL) significantly increased SCE frequency in human PBLs. Based on these results, the study concluded that borax, when used as a food additive, could potentially induce genetic damage. It is important to note that the highest concentration tested in that study (600 µg/mL) was approximately 240 times greater than the boric acid concentrations used in our experiments, which may account for the observed genotoxic effects. These studies support the conclusion that boric acid and related boron compounds are not inherently genotoxic at low to moderate concentrations and may exert significant protective effects against genotoxic agents. However, their safety profile is dose-dependent, and cytotoxicity or genotoxicity may emerge at excessively high concentrations.

The cytokinesis-block micronucleus cytome assay is a sensitive, reliable, and widely adopted method for evaluating the genotoxic, cytotoxic, and cytostatic effects of chemical and physical agents on eukaryotic cells. Among its primary endpoints, the assay quantifies micronuclei-small extranuclear bodies that arise from acentric chromosome fragments or whole chromosomes that fail to incorporate into daughter nuclei during mitosis- serving as indicators of DNA damage or mitotic spindle dysfunction. As a comprehensive multi-endpoint cytogenetic technique, the CBMN-cyt assay enables the simultaneous assessment of several chromosomal instability and cell death, including micronuclei, nucleoplasmic bridges, and nuclear buds. These nuclear anomalies can reflect clastogenic (chromosome-breaking) and aneugenic (chromosome missegregation) mechanisms of action. Elevated frequencies of MN, NBUDs, and particularly NPBs in human peripheral blood lymphocytes have been associated with increased genomic instability and are considered early biomarkers of carcinogenesis [74]. Given its robustness and scope, the CBMN-cyt assay is essential in genetic toxicology. It is also widely employed in screening natural products and synthetic compounds for their potential antigenotoxic or chemopreventive properties. Numerous studies have utilized this assay to evaluate the protective effects of plant extracts, dietary antioxidants, and bioactive natural chemicals, highlighting its value in developing and assessing novel pharmaceutical candidates with genoprotective potential [75].

Mitomycin C, a well-established antineoplastic agent, is known to exert genotoxic and oxidative effects across various mammalian cell types and animal models [50]. In the current study, the cytokinesis-block micronucleus cytome assay application revealed that MMC significantly increased the frequency of micronuclei by approximately 4.15-fold and nuclear buds by nearly fourfold in human lymphocytes, compared to the negative control. Although an increase in nucleoplasmic bridges was observed, it did not reach statistical significance. In contrast, boric acid treatments at concentrations ranging from 0.25 to 2.5 µg/mL did not significantly increase in MN, NBUD, or NPB frequencies. More importantly, co-treatment with BA and MMC produced a statistically significant reduction in MN frequency across all BA concentrations tested. The most substantial protective effects were observed at the two lowest concentrations of BA (0.25 and 0.5 µg/mL), each reducing MN frequency by 77.24%. Even at the highest tested concentration (2.5 µg/mL), BA conferred a notable protective effect, reducing MN frequency by more than 68%. These results underscore the antigenotoxic potential of boric acid in mitigating MMC-induced clastogenicity.

These findings are consistent with a growing body of literature supporting boron-containing compounds' non-genotoxic and chemopreventive properties. For example, Türkez et al. [4] reported no genotoxic effects in the CBMN assay for boric acid, borax, colemanite, and ulexite at 15 to 300 mg/L concentrations. Similarly, other studies have shown that boric acid at 2.5 and 5 mg/L and boron minerals such as borax, colemanite, and ulexite at 5 and 10 mg/L [27, 29, 31, 33] did not significantly increase MN frequencies in human peripheral lymphocytes. Instead, these compounds demonstrated significant protective effects against MN induction by genotoxic agents, including vanadium (5–20 mg/L) [27], titanium dioxide (2–10 µM) [29], and the chemotherapeutic agent paclitaxel (10–20 µg/L) [31]. The protective activity of boron compounds has been largely attributed to their ability to enhance the cellular antioxidant defense system. In animal models, boric acid administered at 3.25 and 13 mg/kg body weight did not induce genotoxicity in rat hepatocytes but significantly attenuated MN formation induced by aluminum exposure [76]. Likewise, Üstündağ et al. [65] demonstrated that boric acid, at 2.5 and 10 µM, did not increase MN frequency in V79 cells but significantly reduced MN formation induced by several genotoxins, including lead chloride, cadmium chloride, vincristine, and 4-nitroquinoline 1-oxide. These effects were associated with an upregulation of cellular antioxidant mechanisms. Further supporting this evidence, Celikezen et al. [62] observed no significant changes in MN frequency following treatment with various concentrations of ammonium tetraborate. Collectively, these findings suggest that boric acid and other boron-containing compounds not only lack genotoxic potential but also confer substantial chemopreventive effects against a variety of DNA-damaging agents. These results reinforce the potential of boric acid as a promising protective candidate for pharmaceutical applications, particularly in combination with genotoxic chemotherapeutics such as MMC, to reduce collateral damage to normal, proliferating cells.

The present study assessed cytotoxicity using multiple cell proliferation indices derived from the same cytogenetic preparations employed for genotoxicity testing. Specifically, the mitotic and replication indexes were evaluated from slides prepared for chromosome aberration and sister chromatid exchange assays. The nuclear division index and cytokinesis-block proliferation index were determined from slides utilized in the cytokinesis-block micronucleus cytome assay. Our findings demonstrated that mitomycin-C significantly decreased the MI compared to the negative control, which can be attributed to its potent cytotoxicity via interstrand DNA crosslinks (ICLs) induction. Even a single ICL event can severely disrupt essential DNA metabolic processes, including replication and transcription, ultimately impairing cell proliferation and survival. Due to this mechanism of action, MMC is extensively employed in chemotherapy, particularly for treating various cancers and dermatological conditions [77]. MMC is most effective during the late G1 and early S phases of the cell cycle [78], and it is known to activate p53 signaling, thereby increasing p53 expression and inducing p53-mediated apoptosis-further contributing to its cytotoxic and anti-proliferative effects [79]. Although boric acid did not significantly increase MI values in MMC-treated human peripheral lymphocytes, a slight but non-significant elevation was observed, suggesting a potential protective effect on cell cycle dynamics. This ameliorative effect may be due to a partial reduction in DNA damage, thereby alleviating MMC-induced cell cycle arrest. In support of these findings, Arslan et al. [3] reported no cytotoxicity in lymphocytes treated with boric acid, ulexite, or borax at concentrations of 2.5, 5, and 10 mg/L for 48 h. Moreover, these boron-containing compounds demonstrated protective efficacy against genotoxic damage induced by 2,3,7,8-tetrachlorodibenzo-p-dioxin. Conversely, previous studies have reported a dose-dependent decrease in MI and RI following 24-h exposure to high concentrations of BA (400–1000 µg/mL) [56]. Similarly, in *Allium cepa* root tip cells, BA reduced MI and increased mitotic aberrations after exposure to 1–4 g/L for 10 and 20 h [34], as well as 20–100 ppm for 5, 10, and 20 h [80]. These effects have been attributed to decreased ATP production and energetic stress on cellular processes [56]. Other cell proliferation indices assessed in this study, including the RI, NDI, and CBPI, also showed no significant alterations upon treatment with BA. These observations align with previous studies reporting that boric acid, borax, colemanite, and ulexite at 5 and 10 mg/L did not significantly affect NDI values in HPLs [33]. Similarly, boric acid alone at concentrations of 2.5 and 5 mg/L did not alter NDI levels but mitigated paclitaxel-induced reductions, restoring NDI values above baseline levels [31]. These findings suggest that at low concentrations, boric acid does not exhibit cytotoxicity and may exert protective effects against chemotherapeutic-induced proliferation arrest, thereby supporting its potential as a chemopreventive agent.

The Comet assay and micronucleus test are extensively employed in genotoxicity and antigenotoxicity studies and in biomonitoring to evaluate DNA damage, repair, and protective mechanisms at the single-cell level. In the present study, hydrogen peroxide, a well-known reactive oxygen species, was utilized as a positive control due to its established capacity to induce oxidative DNA damage. H₂O₂ primarily exerts its genotoxic effects through the Fenton reaction, which generates highly reactive hydroxyl radicals (•OH). These radicals are potent oxidants capable of inducing a range of DNA lesions, including single- and double-strand breaks, base modifications (e.g., formation of 8-hydroxy-2'-deoxyguanosine [8-OHdG]), alkali-labile sites, and DNA–protein crosslinks [38]. In this study, isolated human lymphocytes were exposed for one hour to four concentrations of boric acid (0.25–2.5 µg/mL), both alone and in combination with H₂O₂. As anticipated, H₂O₂ alone caused a substantial increase in DNA damage, evidenced by a five-fold rise in tail intensity compared to the negative control. When applied independently, the two lower concentrations of BA (0.25 and 0.5 µg/mL) did not significantly alter tail intensity, suggesting an absence of genotoxicity at these doses. In contrast, the higher concentrations (1 and 2.5 µg/mL) induced a statistically significant increase in tail intensity, indicating potential DNA damage at these levels, which may not have been effectively repaired within the one-hour exposure period. On the other hand, co-treatment with BA and H₂O₂ significantly reduced H₂O₂-induced DNA damage across all tested BA concentrations. These findings suggest that boric acid possesses a notable protective capacity against oxidative DNA damage, likely attributable to its antioxidant properties. It may act like endogenous antioxidant enzymes such as superoxide dismutase (SOD), catalase (CAT), and glutathione peroxidase (GPX). SOD catalyzes the dismutation of superoxide anions into H₂O₂, which is subsequently detoxified by CAT and GPX into water, thereby mitigating oxidative stress. The BA's observed ameliorative effect may involve the enhancement or mimicry of these enzymatic pathways, contributing to cellular defense against ROS-induced genotoxicity.

Chromosome aberration and micronucleus assays are widely used to assess the frequency of chromosomal and genomic mutations induced and subsequently fixed during previous cell divisions. In contrast, the comet assay directly detects DNA strand breaks in individual nuclei, providing DNA integrity at the moment of analysis. This method is susceptible to early, potentially repairable DNA damage. The primary contributors to increased DNA migration observed in the comet assay include single- and double-strand breaks, single-strand breaks associated with incomplete or delayed excision repair, and alkali-labile sites (ALS)- the latter encompassing DNA lesions such as apurinic/apyrimidinic (AP) sites that convert into strand breaks under alkaline conditions [81]. In the comet assay, isolated cells are directly exposed to test compounds, whereas cytogenetic assays such as CA and MN tests are typically conducted using whole blood cultures. During the lysis step of the comet assay, the cell and nuclear membranes, cytoplasmic contents, and nucleoproteins-including histones-are removed, resulting in a supercoiled, protein-free DNA structure termed a nucleoid. When subjected to electrophoresis, this unprotected DNA relaxes and migrates in the electric field according to the extent of damage, producing the characteristic comet tail. Notably, transient DNA breaks that form as intermediates during active DNA repair processes may also contribute to apparent damage in this assay [81]. The differing experimental conditions between the comet assay and cytogenetic tests may also partly explain variations in the observed genotoxic effects of boric acid. For example, nutrients and other compounds present in the culture medium, such as carbohydrates and vitamins, may interact with BA in the context of intact whole blood cultures, potentially modulating its effects. Conversely, in the comet assay, isolated lymphocytes lack systemic cellular context and may be more vulnerable to direct chemical interactions. In the present study, we hypothesize that such cellular and environmental differences may have contributed to the increased DNA damage observed at the two highest concentrations of BA (1.0 and 2.5 µg/mL) in the comet assay. These results are further supported by a parallel but generally non-significant upward trend in chromosomal aberrations, sister chromatid exchanges, and micronucleus formation. Notably, a significant increase in SCE frequency was observed at 2.5 µg/mL BA after 48 h of exposure, suggesting potential concentration-dependent genotoxicity [81].

The literature broadly supports our findings, particularly about the concentration-dependent dual behavior of boric acid. While BA is known to exhibit antioxidant activity at lower concentrations, higher doses have been associated with pro-oxidant effects. Türkez et al. [4] reported that increasing concentrations of BA significantly suppressed the activities of key antioxidants, including superoxide dismutase, catalase, glutathione peroxidase, and glutathione-S-transferase-alongside reductions in glutathione levels and overall total antioxidant capacity. This impairment of the cellular antioxidant defense system can enhance oxidative stress, ultimately damaging DNA. In our study, the increased comet tail intensity at higher BA concentrations may similarly reflect a shift from protective to damaging effects, likely due to ROS accumulation triggered by the depletion of enzymatic and non-enzymatic antioxidant defenses. These results align with the concept of hormesis, a well-documented biphasic dose–response phenomenon characterized by beneficial effects at low doses and adverse effects at higher doses, typically described by a U-shaped or inverted U-shaped curve. While BA did not induce significant DNA damage at lower concentrations, excessive exposure may have disturbed intracellular redox balance or interfered with DNA integrity, resulting in genotoxic effects [82]. Supporting this interpretation, a study on *Danio rerio* (zebrafish) using the comet assay reported dose-dependent DNA damage following exposure to boric acid and borax at nominal concentrations of 1–64 mg/L over 24 to 96 h [19]. Although tail intensity peaked at 24 h, it declined with longer exposures (48–96 h) yet remained significantly above baseline, suggesting the activation of cellular defense mechanisms such as antioxidant responses, tolerance development, or DNA repair [19]. Further parallel results can be drawn from studies using boron-containing nanomaterials. For instance, boron nitride nanotubes (BNNs) applied to CD34⁺, HeLa, and V79 cell lines showed a reduction in DNA damage after 24 h compared to a 30-min exposure, indicating time-dependent DNA repair at concentrations between 0.5 and 10 µg/mL [83]. Similarly, in *Drosophila melanogaster* larvae, significant DNA damage was observed in the comet assay after 24 ± 2 h of exposure to 2.5 and 5 mM concentrations of boron trioxide nanoparticles and ions but not 0.1 and 1 mM. However, these agents did not induce mutagenic or recombinogenic alterations in the SMART (Somatic Mutation and Recombination Test) wing spot assay. This discrepancy was attributed to the higher sensitivity of the comet assay in detecting transient or repairable DNA strand breaks. In contrast, the SMART test identifies fixed mutations following unsuccessful DNA repair [18]. These findings highlight the nuanced genotoxic profile of boric acid and related boron compounds, emphasizing their dose- and context-dependent behavior. While low concentrations may offer chemoprotective and antioxidant benefits, higher concentrations could risk genomic stability through oxidative stress and DNA fragmentation.

In contrast to findings indicating a dose-dependent genotoxic potential of boric acid at higher concentrations, several studies have demonstrated its safety and protective effects under various experimental conditions. For example, BA did not induce DNA damage in human Sertoli cells (HSeC) in vitro at any concentrations tested, as assessed by the comet assay [84]. Similarly, no significant increase in DNA strand breaks was observed in semen samples obtained from two groups of boron-exposed male workers, suggesting a lack of in vivo genotoxicity in occupational settings [85]. In V79 Chinese hamster lung fibroblasts, 10 µM BA did not result in any observable alteration in DNA damage levels compared to untreated controls. Moreover, when these cells were pretreated with the same concentration of BA, a significant reduction in the genotoxic effects induced by lead and cadmium chloride was observed, highlighting BA’s antigenotoxic capacity in mitigating heavy metal-induced oxidative stress [65]. Comparable protective effects were also observed with other boron-containing compounds. Boric acid, borax, colemanite, and ulexite exhibited no genotoxicity in human peripheral blood lymphocytes or rat bone marrow cells. Furthermore, these compounds effectively antagonize the genotoxic damage induced by aluminum, suggesting a broad chemopreventive potential across different test systems and organisms [33]. Additional evidence from V79 cell experiments showed that although BA alone did not significantly increase DNA fragmentation, pre-incubation with BA markedly decreased hydrogen peroxide-induced DNA damage, further supporting its antioxidant role in cellular defense mechanisms [86]. Reinforcing this perspective, a recent study investigated both elemental boron and boron nitride nanotubes (BNNTs) and found that none of the tested concentrations (0.01, 0.1, 1, 5, and 10 mM) produced a significant increase in DNA damage as measured by comet assay. On the contrary, both compounds significantly reduced the DNA damage and intracellular reactive oxygen species levels induced by potassium dichromate [87]. These findings underscore the context-dependent genotoxic profile of boric acid and related boron compounds. While BA may exhibit pro-oxidant properties at elevated concentrations or under specific conditions, much evidence supports its non-genotoxic and antigenotoxic activity at physiologically and environmentally relevant doses. This dual nature further highlights the importance of dose, exposure duration, and cellular context when evaluating boron-based compounds' safety and therapeutic potential.

Boric acid consistently demonstrated a favorable safety profile and protective properties in other cellular assays as well. For example, BA did not significantly affect cell viability or induce intracellular reactive oxygen species generation in human Sertoli cells (HSeC) [84]. Similarly, boric acid and borax exhibited no cytotoxic effects in both the MTT (3-(4,5-dimethylthiazol-2-yl)−2,5 diphenyltetrazolium bromide) and LDH (lactate dehydrogenase release) assays. In addition, these compounds improved cell viability following exposure to 3-chloro-1,2-propanediol (3-MCPD), a known environmental pollutant. This protective effect enhanced total antioxidant capacity and upregulated antioxidant defense mechanisms [16]. Further supporting these findings, boric acid, borax, and ulexite did not compromise cell viability in MTT assays. More importantly, co-treatment with 2,3,7,8-tetrachlorodibenzo-p-dioxin (TCDD)-one of the most potent environmental toxicants- attenuated the TCDD-induced decrease in cell viability [3]. These boron-containing compounds also preserved cell membrane integrity, as indicated by the mitigation of TCDD-induced lactate dehydrogenase release. Concurrently, they lowered malondialdehyde (MDA) levels, a marker of lipid peroxidation, and enhanced key antioxidant enzymes such as superoxide dismutase and catalase, along with overall total antioxidant capacity[3]. Beyond their direct cytoprotective roles, boron compounds have been shown to modulate intracellular oxidative stress, immune responses, and inflammatory pathways. One of their central mechanisms involves the upregulation of intracellular glutathione, a critical antioxidant that plays a pivotal role in maintaining redox homeostasis and defending against oxidative injury [32].

These findings underscore the significant role of boric acid and other boron-containing compounds in cellular defense mechanisms, particularly through their modulation of antioxidant enzyme activities and capacity to mitigate oxidative stress via multiple biochemical pathways. Boron compounds have modulated membrane receptor functions, potentially influencing transmembrane signaling processes and interacting with specific hormones. Furthermore, they may regulate ionic balance and signaling, contributing to broader physiological responses. In addition to their antioxidant and signaling roles, boron compounds are believed to participate in key metabolic processes. These include the synthesis and degradation of essential minerals such as calcium and phosphate, regulation of hormone activity-including 1,25-dihydroxycholecalciferol (the active form of vitamin D)-and the facilitation of protein and lipid metabolism through the activation of various enzymatic steps [88]. This multifunctional involvement suggests that boron protects against genotoxic and oxidative insults and is a potentially vital micronutrient in maintaining cellular and systemic homeostasis.

## Conclusion

When evaluating the protective potential of compounds against genotoxic agents, various treatment regimens-such as pretreatment (administered before exposure to neutralize potential damage), post-treatment (applied following exposure to facilitate repair), and co-treatment (simultaneous application to achieve both preventive and reparative effects)-are commonly employed. The present study utilized a co-treatment strategy wherein boric acid was administered concurrently with the genotoxic agents mitomycin C and hydrogen peroxide. The findings demonstrated that boric acid, at the investigated concentrations, did not exhibit significant genotoxic, DNA-damaging (except at higher concentrations) or cytotoxic effects in vitro. Moreover, BA significantly mitigated the genotoxic impact of MMC and H₂O₂ across multiple endpoints-including aberrant cell frequency, chromosome aberrations per cell, micronucleus formation, and sister chromatid exchanges in cultured human peripheral blood lymphocytes and comet assay in isolated lymphocytes. This protective potential was especially pronounced at lower concentrations of BA, suggesting a dose-dependent cytoprotective effect. These effects are likely mediated through inhibiting reactive oxygen species generation and activating the antioxidant defense system, aligning with the known redox-modulating properties of boron compounds. Accordingly, the data support the potential utility of low-dose boric acid as a chemoprotective adjunct in antineoplastic therapies, potentially reducing the genotoxic side effects of chemotherapeutic agents. However, further research is warranted to elucidate the precise mechanistic basis and evaluate its broader therapeutic relevance. Future studies should explore a wider range of boric acid concentrations, diverse in vivo models, and additional genotoxicity assays to comprehensively assess the role of boric acid in cancer prevention or supportive care during chemotherapy.

## Data Availability

No datasets were generated or analysed during the current study.
